# Structural Versatility of Bicellar Systems and Their Possibilities as Colloidal Carriers

**DOI:** 10.3390/pharmaceutics3030636

**Published:** 2011-09-14

**Authors:** Barbosa-Barros Lucyanna, Rodríguez Gelen, Cócera Merce, Rubio Laia, López-Iglesias Carmen, de la Maza Alfons, López Olga

**Affiliations:** 1 Departament de Fisicoquímica, Facultat de Farmàcia, Universitat de Barcelona, 08028 Barcelona, Spain; 2 Departament de Tecnologia Química i de Tensioactius, Institut de Química Avançada de Catalunya (I.Q.A.C.), Consejo Superior de Investigaciones Científicas (C.S.I.C.), C/Jordi Girona 18-26, 08034 Barcelona, Spain; 3 Centres Científics i Tecnològics, Universitat de Barcelona, Parc Científic de Barcelona, C/Josep Samitier 1-5, 08028 Barcelona, Spain

**Keywords:** bicellar systems, bicelles, ceramides, nanostructures, colloids, freeze fracture electron microscopy, phospholipids, skin, stratum corneum, transition temperature

## Abstract

Bicellar systems are lipid nanostructures formed by long- and short-chained phospholipids dispersed in aqueous solution. The morphological transitions of bicellar aggregates due to temperature, composition and time variations have been revised in this work. To this end, two bicellar systems have been considered; one formed by dimyristoyl-phosphatidylcholine (DMPC) and dihexanoyl- phosphatidylcholine (DHPC) and another formed by dipalmitoyl-phosphatidylcholine (DPPC) and DHPC. The relationship between the magnetic alignment, the morphology of the aggregates and the phase transition temperature (*T*_m_) of lipids is discussed. In general terms, the non-alignable samples present rounded objects at temperature below the *T*_m_. Above this temperature, an increase of viscosity is followed by the formation of large elongated aggregates. Alignable samples presented discoidal objects below the *T*_m_. The best alignment was achieved above this temperature with large areas of lamellar stacked bilayers and some multilamellar vesicles. The effect of the inclusion of ceramides with different chain lengths in the structure of bicelles is also revised in the present article. A number of physical techniques show that the bicellar structures are affected by both the concentration and the type of ceramide. Systems are able to incorporate 10% mol of ceramides that probably are organized forming domains. The addition of 20% mol of ceramides promotes destabilization of bicelles, promoting the formation of mixed systems that include large structures. Bicellar systems have demonstrated to be morphologically stable with time, able to encapsulate different actives and to induce specific effects on the skin. These facts make bicellar systems good candidates as colloidal carriers for dermal delivery. However, water dilution induces structural changes and formation of vesicular structures in the systems; stabilization strategies have been been explored in recent works and are also updated here.

## 1. Introduction

Bicelles are lipid nanostructures formed by a long and a short chain phospholipid dispersed in aqueous solution [1,2]. These aggregates have been used as membrane models for nuclear magnetic resonance (NMR) structural studies of membrane biomolecules [3]. Depending on the composition and the long/short chain phospholipid molar ratio (*q*), these structures may display a bilayered discoidal morphology alignable in magnetic fields at temperatures above the long chain phospholipid phase transition (*T*_m_). The total lipid concentration of bicelles is also an important factor, since the dilution of these aggregates promotes important morphological transitions [4]. The disks formed by the combination of the dimyristoyl phosphatidylcholine (DMPC) constituting the flat bilayer, plus the dihexanoyl phosphatidylcholine (DHPC) stabilizing the rim of the structures have been the most applied systems for these kind of studies [5-7]. Some works have shown the morphology and phase diagram of DMPC/DHPC bicelles to be very complex. In general, it is accepted that bicelles for which the long chain to short chain phospholipid molar ratio is between 2.8 and 6.5 will spontaneously align in a magnetic field, such that the bilayer plane is parallel to the magnetic field. The short chain amphiphile (DHPC) plays a slightly different role in such aligned aggregates; in most cases, DHPC-rich torroidal defects are believed to be scattered throughout an extended bilayer resembling a perforated lamellar morphology [8,9]. The DHPC-rich defects lend sufficient mobility to the overall aggregate that spontaneous alignment can occur.

One of the aims of this review is to understand the morphology and phase behavior of bicelles over a wide range of temperatures and compositions. This is interesting considering that bicelles are able to mimic particular types of cell membranes [10-14]. The formation of bicelles with different phospholipids as well as the inclusion of cholesterol, ceramides (Cer), cardiolipin and polyethylene glycol (PEG) lipids in the systems has been also recently reported [15-19]. Thus, bicelles are replacing lipid based liposomes and surfactant based micellar systems as models to study membrane proteins.

Bicelles exhibit an intermediate morphology between lipid vesicles and the classical mixed micelles, combining some of the attractive properties of both of these model membrane systems. Like micelles, bicelles are non-compartmentalized, optically transparent, and effectively mono-disperse. Consequently, it is much easier to achieve homogeneous mixing in bicelles than in lipid vesicles. On the other hand, bicelles, in contrast with the classical mixed micelles, have not surfactants in their structure and maintain some key bilayer properties that are absent in the latter systems [20]. Although both liposomes and micelles have often been used in skin treatment [21,22] their application to date has been extensively debated. In fact, the large size of liposomes makes its penetration in the skin difficult, and micelles usually promote skin irritation due to their surfactant composition. In this field, the bicellar systems seems to offer advantages over liposomes because of its dimensions (diameters in the range of 10–50 nm and thickness about 4–6 nm [1]), small enough for passing through the stratum corneum SC lipid lamellae; and over micelles due to its bilayered structure and exclusively lipid composition [23,24].

This revision includes studies of the morphological transformations of specific bicellar systems due to the changes in the composition, temperature and time after preparation. Two bicellar systems have been revised; a classic one formed by dimyristoyl phosphatidylcholine (DMPC) and dihexanoyl phosphatidylcholine (DHPC) and another formed by DPPC dipalmitoyl phosphatidylcholine and also by the DHPC. Besides, the influence of different Cer in these systems has also been described. In general, the alignment of the systems has been studied using ^31^P-NMR. The characterization of the structural changes produced in bicelles by effect of different factors is focused, combining different techniques such as: small-angle X-ray scattering (SAXS); dynamic light scattering (DLS) and freeze fracture electron microscopy high-pressure freezing (FFEM-HPF).

Taking into account the specific characteristics of bicelles and their high versatility, the use of these lipid nanostructured systems as colloidal carrier should be considered.

## 2. DMPC/DHPC Bicellar Systems

### 2.1. Relationship between the Changes in the Physico-Chemical Characteristics of Bicelles and the Temperature

The classical description of bicelles as discoidal objects formed by a DMPC bilayer closed in the edges by DHPC molecules has been well accepted for non-aligned samples below their main phase transition temperature (*T*_m_). However, the description above the *T*_m_ was in the past extensively discussed, where the magnetically alignable phase was thought to be formed by shaped-disks [1-4,25].

In the last years diverse studies have shown the morphology and phase diagram of DMPC/DHPC bicelles to be very complex and under some debate [26-29]. Nieh and co-workers proposed a model for bicelles with and without lanthanide cations (Ln^3+^), which is commented on in the following paragraph [9,27].

At temperatures below the T_m,_ of the DMPC, the resulting bicelles were shaped disks [9]. As the temperature rose from the gel to liquid crystalline phase in the presence of Ln^3+^ the bicelles fused together in an end-to-end manner to form lamellar sheets with perforated holes that were lined with DHPC. Further temperature increase caused phase separation with the formation of DHPC-rich mixed micelles and DMPC-rich oriented lamellae, and the DHPC-rich mixed micelles became incorporated into the oriented bilayers at even higher temperatures [30]. In the absence of Ln^3+^, bicelles were disk-shaped in the gel phase, and chiral nematic in the liquid crystalline phase, which were described as wormlike micelles, and at higher temperatures were multilamellar vesicles [27]. Additionally, recent studies have demonstrated that inclusion of small amount of charged lipids eliminated the appearance of the worm-like or ribbon phase [31] Hereafter, the use of the term bicelle in most studies refers only to the sample composition and not to the disk-shaped morphology. A comparative study of alignable and non-alignable bicelles associated to the temperature changes of the system could be helpful to clarify the morphological transitions involved in this process.

Our group has recently published studies [32,33], in which high pressure freeze fixation and freeze fracture electron microscopy techniques [34,35] were combined with the ^31^P-NMR to correlate the magnetic alignment of DMPC/DHPC bicelles, morphological transitions and the changes in the temperature of the system. Two lipid molar ratios (*q* = 2 and *q* = 3.5) were investigated. Direct observations showed that *q* = 2 bicelles (S-q2) were very fluid and transparent at 20 °C, but as the temperature rose, the sample became viscous with a gel-like appearance maintaining the transparency until 60 °C. When q was 3.5 (S-q3.5) the system became fluid and transparent only at temperatures below DMPC T_m_, (around 24 °C) [36]. At 25 °C the sample became viscous, at 30 °C it was viscous and slightly milky and from 40 °C to 60 °C was milky and fluid.

**Figure 1.**
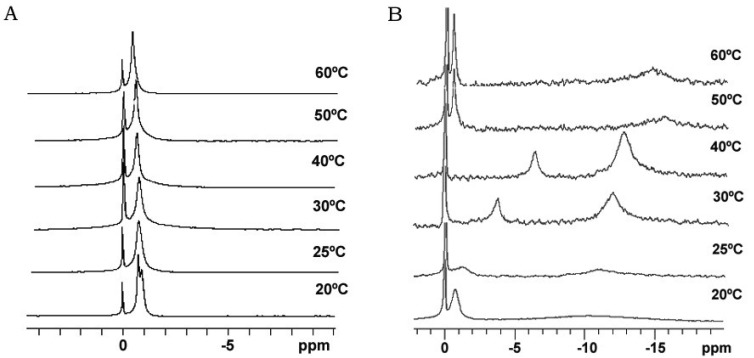
^31^P NMR spectra under ^1^H decoupling as a function of temperature of the S-q2 system (left column) and the S-q3.5 system (right column). The temperature is indicated in the figure and was increased smoothly starting from 15 °C with equilibration times of 15 min between different temperatures—the reference resonance at 0 ppm arises from 85% phosphoric acid. Water content in both systems was 80%.

Bicelle alignment was studied using ^31^P-NMR technique. Figure 1 shows the spectra for (S-q2) and (S-q3.5) bicelles in a temperature range from 20 to 60 °C [32]. The reference peak is shown at 0 ppm. The spectra of the S-q2 (Panel A) showed isotropic behavior at all temperatures. At 20 °C the spectrum was a doublet near −1 ppm reflecting the slightly different environments of the DMPC sites (bilayer) and DHPC sites (rim) on the bicelle structure [37]. At 25 °C, these peaks overlapped. The single peak slightly shifted downfield with increasing temperature until 60 °C, showing a characteristic behavior of non-alignable phospholipid mixtures [38,39].

The spectra of S-q3.5 (Figure 1, Panel B) presented considerable variations depending on the temperature. At 20 °C a single resonance peak was shown at −0.77 ppm and a broad protuberance was seen around −10 ppm, suggesting that the sample had some orientation with respect to the magnetic field. The presence of two well differentiated resonances in bicellar samples has been reported by Gaemers and Bax as a result of the alignment occurring with the bilayer normal oriented perpendicular to the magnetic field [40]. The high-field resonance would correspond to DMPC oriented bilayers and the low-field resonance to DHPC forming disks or hole edges [6]. Just above the DMPC Tm, at 25 °C, the spectrum showed two broad resonances at −1.19 and −10.82 ppm. From this temperature, the observed resonances move towards the right and the lines became more intense, indicating an improvement of the magnetic alignment that reaches a maximum at 40 °C. The integration of the high and low-field peaks gave a relative intensity of 3.3. This value was close to the q ratio of this sample 3.5, confirming that the downfield peak corresponded to the DHPC and the upfield to the DMPC. The gradual upfield shift of DMPC peak reflected an increase of the bilayer order with respect to the magnetic field and the upfield shift of the DHPC peak was related to the increasing presence of DHPC in the DMPC bilayer. That is, the rise of temperature promoted the miscibility of the DHPC molecules in the DMPC bilayer that resulted in the migration of the DHPC molecules from the edges of the bicelles to the bilayer area. Hence, the absence of enough DHPC molecules to fill the bicelle edges led to the fusion of bilayers, which increased the bicellar diameters and improved the alignment until a certain point. From 50 °C the sample orientation was lost and a typical isotropic low field peak around −0.54 ppm and a broad protuberance around −15 ppm were present. According to Triba and co-workers [37] this change on the spectra was characteristic of a phase transition from bicellar aggregates to bigger structures with slow motion. To elucidate the morphological characteristics of these bicellar systems electron microscopy experiments were performed. To this end, alignable (S-q3.5) and non-alignable (S-q2) samples, below the DMPC T_m_ and above this temperature were analyzed. Bicelles were high pressure frozen (HPF) at −150 °C from sample temperature of 20 °C and 40 °C. These temperatures were chosen to compare the morphology of these alignable and non-alignable samples, to further correlate with the corresponding ^31^P-NMR data.

**Figure 2.**
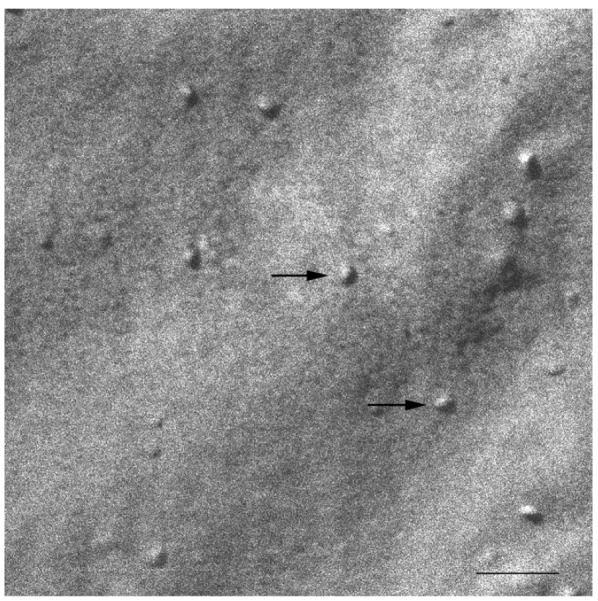
Electron micrograph of the S-q2 system at 20 °C. The arrows indicate the small bicelles of about 20 nm. Bar = 100 nm.

**Figure 3.**
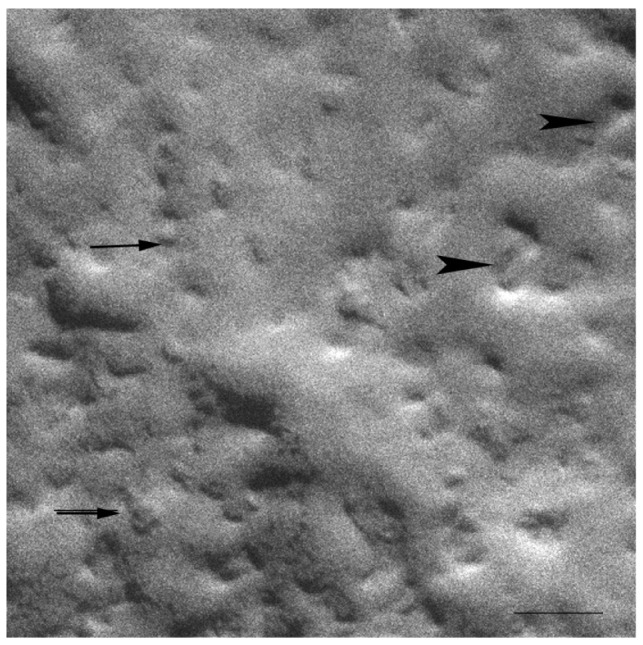
Electron micrograph of the S-q3.5 system at 20 °C. The arrows denote disks viewed face-on and the arrow heads denote disks viewed edge-on. Bar = 50 nm.

The HPF method was developed to allow for the freezing of hydrated material from a defined state [35]. The great advantage of this method is the non-requirement of sample pre-treatments, which frequently leads to the formation of artefacts. The freeze fracture replicas were visualized mainly by a cryo-SEM technique [33]. Replica cleaning for TEM is tedious and sometimes difficult, large replicas tend to disintegrate during cleaning and the field of view is restricted by the grid bars. These problems are circumvented by cryo-SEM [41], in which no replica cleaning is requested. In SEM, the sample does not have to be transparent to the electron beam, and bulk samples can be analyzed. Using the combination of HPF and cryo-SEM, samples difficult to clean for the TEM were imaged and large areas were investigated. All investigated samples were free of visible ice crystal artefacts. The cryo-SEM images for S-q2 and S-q3.5 samples are shown in Figures 2 and 3, respectively.

At 20 °C, temperature below T_m_, small aggregates were visualized in both cases (Figures 2 and 3). Figure 2 shows small rounded aggregates with diameters around 20 nm (arrows). In Figure 3 the aggregate diameters of S-q3.5 were about 40 nm (slightly larger than those of S-q2), being also visualized a discoidal structure shape. Two different zones in the discoidal structures are observed. The arrows indicate aggregates with face-on and the arrow heads point to aggregates with edges-on.

Comparison of these images with the corresponding ^31^P-NMR spectra, revealed a relation between the aggregate structure and its magnetic orientation. The rounded shape aggregates of the S-q2 image did not allow for magnetic alignment, whereas the two well differentiated areas of the disks visualized in the S-q3.5 micrograph accounted for the onset of orientation detected in Figure 1B by the presence of the two separated resonances.

**Figure 4.**
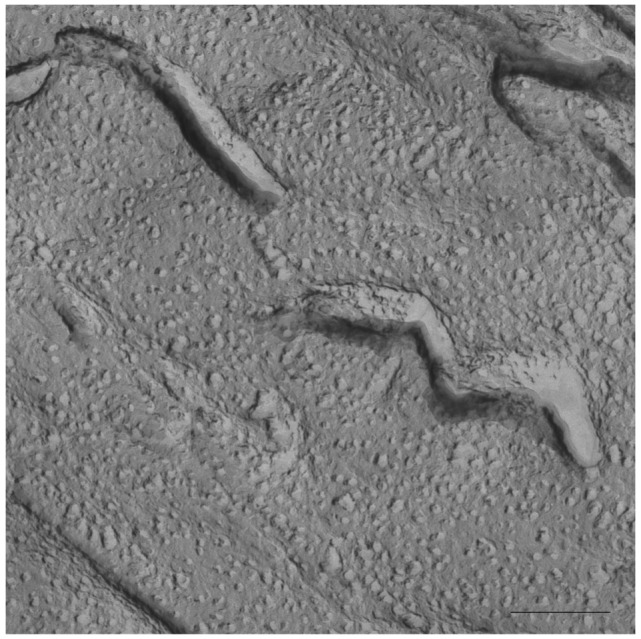
Electron micrograph image of the S-q2 system at 40 °C showing isolated elongated aggregates. Bar = 1.00 μm.

At temperature of 40 °C large aggregates were observed for both in the S-q2 and the S-q3.5 systems. Figure 4 shows an image for S-q2 system with elongated aggregates with about 2000 nm. Similar results have been reported by vam Dam and co-workers for DMPC/DHPC systems at *q* = 2, low concentrated samples (*c*_L_ = 3%) at temperatures of 36 °C and 46 °C using a cryo-TEM technique [42].

**Figure 5.**
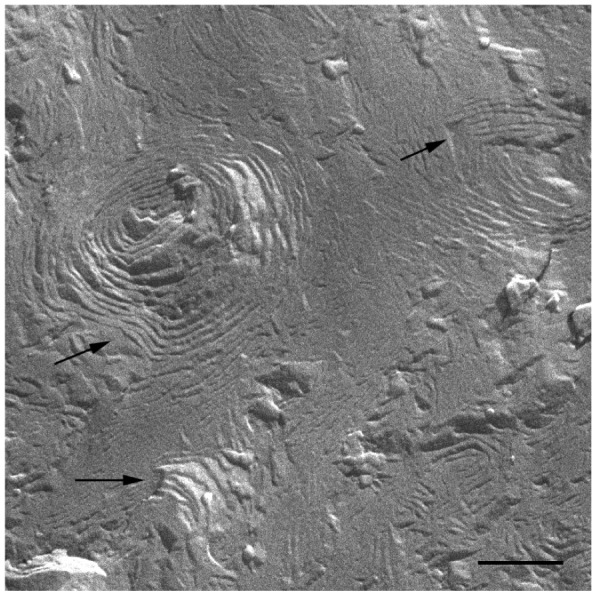
Electron micrograph image of the S-q3.5 system at 40 °C showing stacked bilayer sheets (arrows). Bar = 500 nm.

In Figure 5 a cryo-SEM image of the sample S-q3.5 at 40 °C is displayed in which extended areas of stacked lamellar sheets (arrows) were visualized. The presence of multilamellar vesicles was also detected.

**Figure 6.**
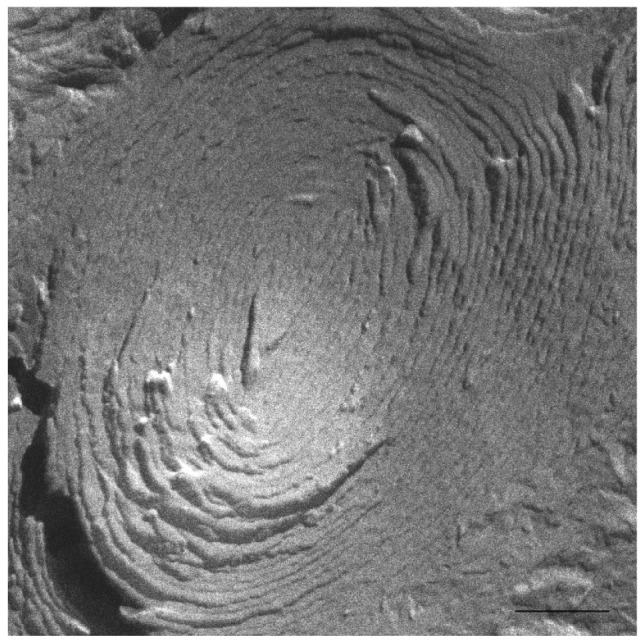
Electron micrograph showing a multilamellar vesicle present in the S-q3.5 system at 40 °C. Bar = 250 nm.

Figure 6 depicts a detail of one of these multilamellar vesicles with a size around 1000 nm. The large structures observed in these micrographs accounts for the milky appearance of this sample. This lamellar phase probably represents the most ordered phase of this sample since the best alignment in ^31^P-NMR experiments was obtained at this temperature.

The current discussion about the morphology of the DMPC/DHPC aligned aggregates may be summarized in two hypotheses. Both coincide in the morphological transitions of the aggregates from disks to elongated micelles, perforated lamellar sheets and mixed multilamellar vesicles. Also, both hypotheses take into account the initial increase of sample viscosity and the subsequent drop in this viscosity caused by the continuous increase of temperature. Nevertheless, they diverge on the morphology of the structures when sample shows magnetic alignment. One of the theories defends that elongated aggregates correspond to the morphology of the magnetic aligned phase and are present when the sample viscosity increases [43]. The other, claims that the perforated lamellar sheets corresponded to the morphology of the magnetic aligned phase. The formation of these lamellar structures would take place when the sample viscosity drops and the sample appearance is milky [5,6]. Moreover, in a deep ^31^P-NMR study Triba and co-workers [37] admitted the compatibility of both elongated and perforated lamellar structures with the NMR aligned spectra, although they did not disprove the discoidal structure as possible in the aligned phase.

The results obtained by our group combining HPF, FFEM and ^31^P-NMR techniques [32] are in accordance with some points of the mentioned theories. In the experiments with the sample S-q2 we observed the increase of sample viscosity when the sample was heated. The cryo-SEM images of this sample at 40 °C showed elongated structures, as described by Harroun [43] (Figure. 4). Nevertheless, this sample did not align in magnetic fields (Figure 1A). At this q ratio, the proximity of the DMPC and DHPC molecules in the elongated structures formed at 40 °C as well as in the spherical objects formed at 20 °C did not allow for an anisotropic environment. Hence, the two phosphorus signals in ^31^P-NMR overlap and a single resonance is seen. In this way, although the increase of sample viscosity can be attributed to the formation of elongated aggregates, these structures did not correspond to the morphology of the magnetic aligned phase.

On the other hand, evidence of the morphology of aligned aggregates was obtained by the analysis of the S-q3.5 micrographs. This sample also presented an increase of the viscosity when the temperature raised, but when the best alignment was detected (40 °C), the sample had a milky appearance. The cryo-SEM micrographs at this temperature (Figures 5 and 6) showed areas plenty of stacked bilayer sheets and some multilamellar liposomes. The closing up of the bilayer sheets in vesicles is the result of a spontaneous process probably caused by the reduction of the energetic cost of bending by the effect of temperature. The existence of a mixed system accounts for the broad peaks obtained in the spectrum at 40 °C. The multilamellar vesicles probably interfere in the perfect alignment of the structures which would produce narrow resonance peaks. In any case, the segregation of DMPC and DHPC in the S-q3.5 aggregates accounts for the orientation of this system in the magnetic field. Even below T_m_, the discoidal shape of these aggregates (Figure 3) caused the onset of orientation reflected in the appearance of the broad resonance at −10 ppm in the NMR spectra at 20 °C (Figure 1B**)**. The improvement of the alignment of this sample was observed with the increase of temperature reaching a maximum at 40 °C, temperature at which stacked lamellar sheets were mainly observed. From 50 °C, the lost magnetic alignment and the resonance signal at −15 ppm probably indicated the complete closing up of the aligned bilayer sheets into isotropic vesicles. From these data it may be assumed that the combination of NMR and HPF, FFEM techniques was appropriated to evaluate the relation between the magnetic alignment and the morphology of the two bicellar systems revised.

As for the isotropy of the non-alignable sample S-q2, morphological evolutions from spherical to elongated aggregates were reported, verifying that despite of their growth, these elongated aggregates were not able to orient in magnetic fields. As for S-q3.5 these samples presented anisotropy for magnetic orientation even below *T*_m_, given that the morphology of the aggregates allows distinct phosphorus environments. As the temperature increased the sample achieved best alignment, and the morphology of the aggregates evolved from disks to lamellae. At the temperature of the best magnetic alignment, the aggregates were formed by stacked bilayer sheets and multilamellar liposomes. The present data could be very useful to modulate the alignment of systems in magnetic fields and therefore to optimize the use of bicellar systems as membrane mimics.

### 2.2. Stability of Bicelles against the Time

The temporal stability of these aggregates has been analyzed using ^31^P-NMR, FFEM, HPF, Small-Angle X-ray Scattering (SAXS) [44-46], and Dynamic Light Scattering (DLS) techniques.

**Figure 7.**
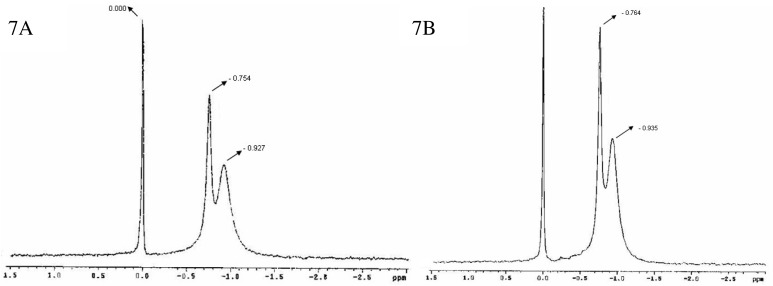
P NMR spectra spectra at 20 °C for DMP/DHPC bicelles (*q* = 2 and 80% water content) 24 h **(A)** and 14 days **(B)** after preparation.

Figure 7 shows the ^31^P-NMR spectra for bicelles 24 h (7A) and 14 days (7B) after preparation [47]. A doublet was observed with peaks around −0.754 and −0.927 ppm (7A) and −0.764 and −0.935 ppm (7B) respectively, in addition to the reference peak sited at 0.000 ppm. As aforementioned, this spectral appearance has been previously reported by Triba and co-workers for small disks in rapid rotation [37]. The reproducibility of the bicelles characteristic resonances after 24 h and 14 days indicated a good structural stability of the sample at least for 2 weeks.

**Figure 8.**
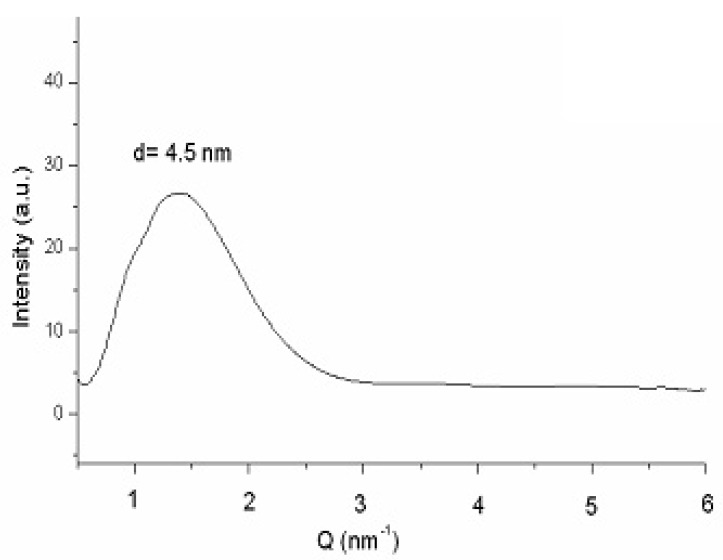
Small angle X-ray scattering (SAXS) curve at 20 °C of bicelles (*q* = 2, 80% water content) 24 h after preparation.

Figure 8 plots the SAXS curve of bicelles 24 h after preparation [47]. The fact that no differences were detected between this scattering curve and that obtained 14 days after preparation (data not shown), also corroborates the stability of the system. The lamellar repeated distance “d” was estimated from the analysis of the peak by the Bragg's law and was attributed to the bilayer thickness in a similar way that studies with liposomes and other bilayer models [48]. A repeat distance value *of d* = 4.5 nm was obtained in both spectra (24 h and 14 days after preparation), indicating that no alteration in the bilayer occurred during the 14 days of experiment.

**Figure 9.**
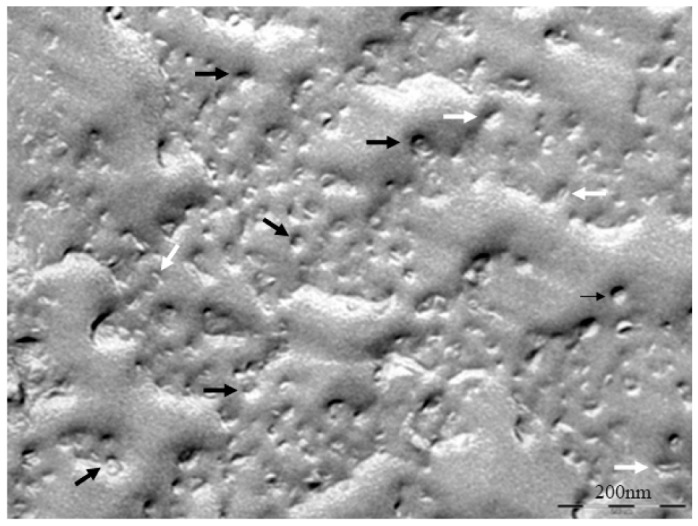
Electron micrograph of DMPC/DHPC *q* = 2 bicelles. The image depicts a zone full of bicelles. Black arrows denote disks viewed face-on and white arrows denote disks viewed edge-on.

The size distributions curves obtained by DLS exhibited a monomodal distribution with a hydrodynamic diameter (HD) of 15.9 nm and a polydispersity index (PI) of 0.211, 24 h after preparation [47]. Values for the same sample 14 days after preparation resulted very similar: (HD = 16.3 nm and PI = 0.303). The HD obtained by DLS is that of a hypothetical hard sphere that diffuses with the same speed as the particle under experiment. As the bicelles present a flat appearance, the values of particle size obtained may be considered as an estimation of the structure's dimension. The particle size values obtained showed quite good agreement with the structure sizes visualized by SEM. Figure 9 shows SEM micrograph of the S-q2 bicelles at 20 °C, 14 days after preparation. The arrows indicate the small rounded bicelles of about 20nm (Bar = 100 nm) similar to those shown in a SEM micrograph of bicelles S-q2 after preparation also at 20 °C (Figure 2). Similar structures have been reported by other authors using other electron microscopy techniques [32,34,36,49,50].

In resume, the physicochemical characterization of the bicellar system S-q2 indicated structural stability at least for 14 days after preparation.

## 3. DPPC/DHPC Bicellar Systems

In the preceding section we revised some specific physico-chemical properties of the conventional DMPC/DHPC bicelles at different conditions. In the present section bicelles formed by 1,2-dipalmitoyl-sn-glycero-3-phosphocholine (DPPC) and the conventional DHPC have been updated. Furthermore, in these systems the q ratio has been also adjusted to 3.5 in order to diminish the concentration of DHPC and total lipid concentration (CL) was adjusted to 20%. Some reasons are considered to introduce these changes. One reason is based on the difference in the *T*_m_ values (24.1 °C for DMPC and 41.5 °C for DPPC and [51,52]) despite the increase of only two carbons the long-chained lipid (from 14 to 16). This fact results in significantly different polarities above and below their *T*_m_ [53]. Furthermore although bicelles formed by both lipids exhibit similar dynamical behavior, the DPPC lipid acyl chain displayed a somewhat lower degree of mobility evidenced by higher generalized order parameters throughout the acyl chain [54]. On the other hand, thinking in the application of bicellar systems as colloidal carriers the preservation of the shape and size of bicelles would be a requirement. Hence, it is necessary that Tm of lipids building bicelles exhibit higher values than the experimental temperature (for *in vivo* application about 37 °C). The increase in the q value to 3.5 (diminution in the relative proportion of DHPC) was chosen in order to diminish the surfactant character of the lipid mixture thus making the model more appropriate for *in vivo* studies.

In the present section we revised the dimensions and morphology of DPPC/DHPC bicelles and the bicelle-vesicle transition that takes place by the effect of dilution. A dilution of the systems would be desirable for purposes of the use of bicelles as colloidal delivery systems. The physico-chemical characteristics of this new bicellar system have been characterized using ^31^PNMR, SAXS, DLS, and FFEM techniques.

### 3.1. Dimensions and Morphology of the DPPC/DHPC Bicellar Systems

**Figure 10.**
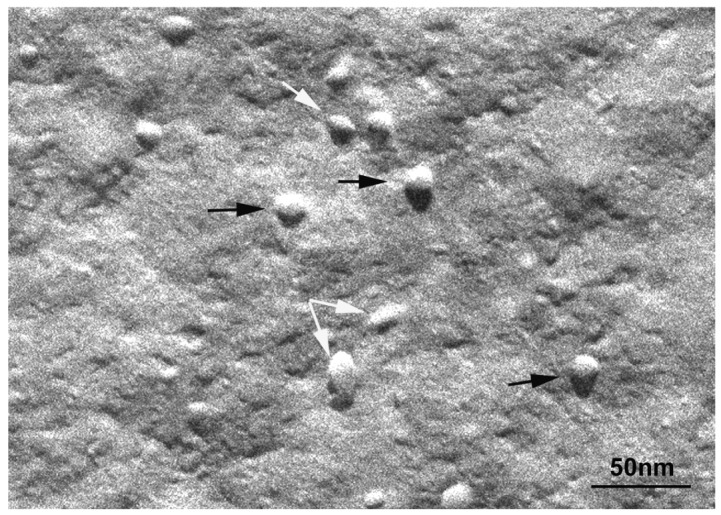
Micrograph of DPPC/DHPC bicelles at 37 °C. The black arrows denote the disks in face-on disposition and the white arrows correspond to the disks in edge-on.

The characterization of the DPPC/DHPC bicelles is in general performed following the same methodology and techniques as those used to characterize the DMPC/DHPC bicelles (section 2.1.) [32,55]. Applying the form factor squared of the simplified Gaussian model to SAXS a bilayer thickness value (d_B_ = 5.4 nm) was obtained [32,55]. The size distribution curve obtained by DLS shows a HD value of 11.3 nm and a PI of 0.072. This particle size provides a relative measurement of the structure dimensions. Therefore, this data should be interpreted taking into consideration also the electron microscopy images, in which a direct visualization of the bicellar structures is obtained. Figure 10 shows a cryo-SEM image at 37 °C, in which small discoidal aggregates of about 15 nm in diameter were observed. In this image, the face (black arrows) and the edges (white arrows) of the disks are visualized. As a consequence, this result shows quite good agreement with the DLS data despite the different resolution of each technique.

### 3.2. Bicelle to Vesicle Transition States Obtained by Dilution

**Figure 11.**
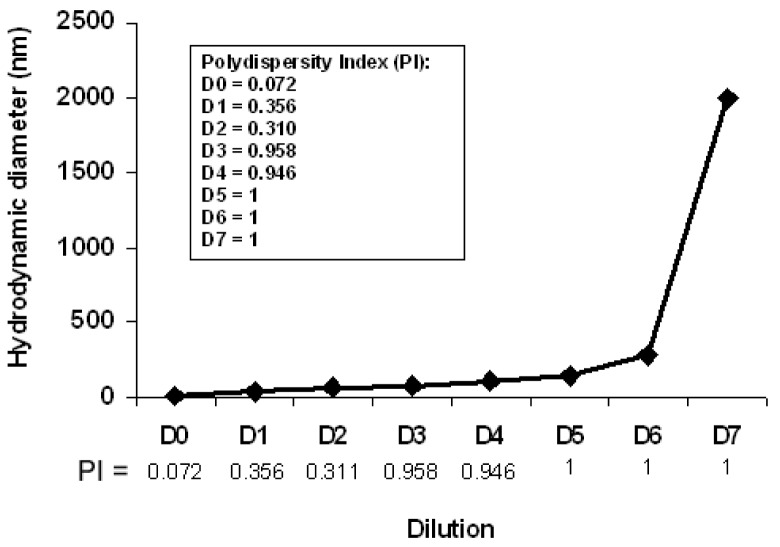
Evolution of the average particle size of the DPPC/DHPC bicelles with *q* = 3.5 by the effect of dilution as measured by DLS. The polydispersity indices are shown in the inset.

To monitor the effect of dilution on the structure of the bicelles, 2 mL of a bicelle sample (*q* = 3.5, and lipid concentration of 20%) was sequentially diluted with de-ionized water in seven steps (D1-D7) to obtain concentrations from 5% to 0.07%. Twenty-four hours after the dilutions, the HD and PI of each diluted sample were measured by DLS at 37 °C. An overview of this process is plotted in Figure 11.

The DLS curves of the diluted samples showed that the HDs of the structures increase upon dilution from values of about 11.3 nm (assigned to bicelle disks) to large size aggregates bigger than 1 μm.

**Figure 12.**
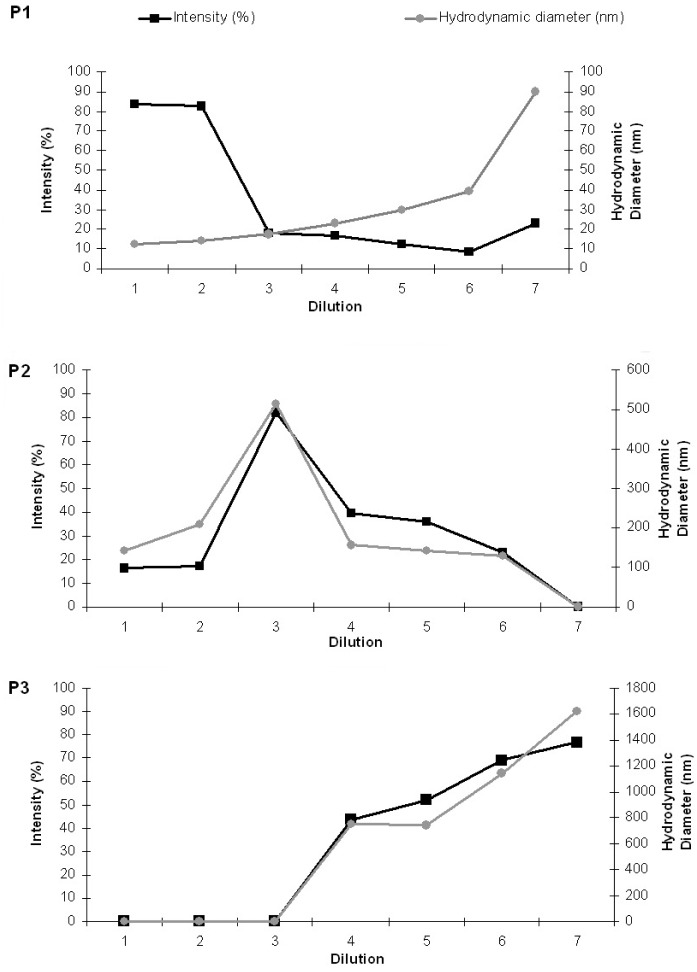
Evolution of the DLS scattered intensity and hydrodynamic diameter of the DPPC/DHPC bicelles upon dilution. P1 denotes the evolution of particles with sizes in the range of 10–100 nm (**A**). P2 denotes intermediate sized particles with diameters around 100–500 nm (**B**) and P3 indicates the bigger particles >500 nm (**C**).

For better understanding of the results, aside from the evolution of the average particle sizes, a detailed analysis of the scattering intensity was performed. The particle sizes obtained were separated in three groups: P1 included particle sizes in the range 10–100 nm, P2 included intermediate particle sizes ranging from 101 to 500 nm, and P3 included particle size values >500 nm. The HDs and the percents of light scattered of each group area plotted separately as a function of the sequential sample dilution in Figure 12. It is noteworthy that the intensity for small bicelles (P1) diminished with dilution, whereas the HD increased (Panel A). Intermediate aggregates (P2 in panel B) were present from the first dilution on and display a maximum of intensity and size at D3. From D4, the intensity of these aggregates diminishes and bigger structures are detected (P3 in panel C), coexisting with P1 and P2 until D6. The high PI values (shown in Figure 11 from D3 onward) are explained by the high variety of aggregates present in these samples.

It is noteworthy that the percent of the intensity curves does not represent the percentage of the structures present in the systems. In DLS, the HD is calculated from the intensity of the scattered light and gives information about the different particles present in the sample. From the Rayleigh approximation follows that the intensity is proportional to d^6^ (d is the particle diameter); thus, the contribution of the light scattered from small particles is relatively small when compared to that of large particles that scatter much more light. Hence, the intensity curves obtained indicate the appearance of bigger aggregates by dilution but do not accurately quantify them. In general, the percent intensity of these structures and their HD increase in each dilution (Figure 12).

**Figure 13.**
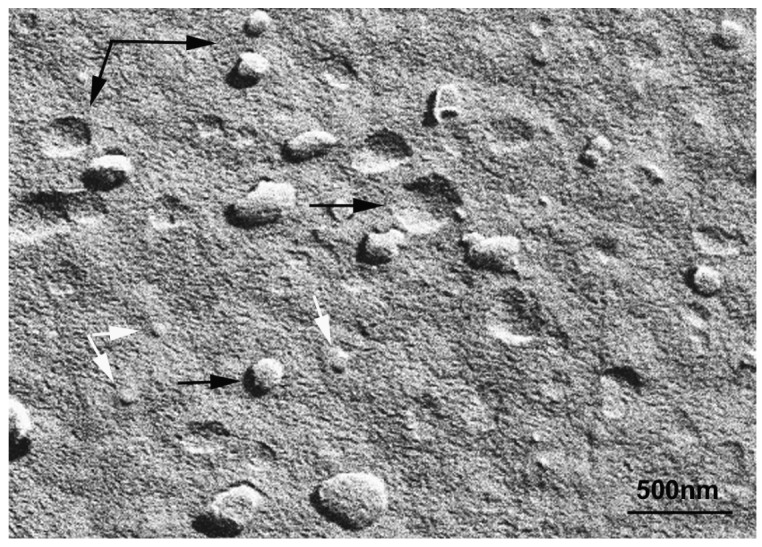
Micrograph of the diluted DPPC/DHPC bicelles (D5) at 37 °C. The black arrows denote vesicle structures of about 200–500 nm and the white arrows point to bicellar structures.

The coexistence of these big aggregates with the small bicelles is detected until the last dilution performed (D7). In order to investigate the morphology of these systems, the diluted bicellar solutions were analyzed by microscopy. A representative cryo-SEM image of the sample D5 is shown in Figure 13. This image reveals the presence of vesicles of about 200–500 nm (black arrows) together with small bicelles (white arrows). Comparing this micrograph to that of the original system (Figure 10), the increase of structures size and the variety of bigger aggregates in the sample are noteworthy. This result is fully consistent with DLS data corroborating the transition of bicelles from disks to vesicles by the effect of dilution. This transition took place by progressive steps that implied the coexistence of different aggregate structures in the medium.

In general terms, although the classical bicelle model formed by DMPC and DHPC has been successfully used, another bicelle systems made up of lipids with different acyl chains, backbones, or headgroups have offered alternatives for different studies [12,16,56-58]. As aforementioned, the DPPC is one of the most studied lipids for bilayer models due to its longer acyl chains, and the bilayer thickness [59] and has the additional advantage of having a higher value of *T*_m_ [60-62].

The phase transitions that occurred at *T*_m_ involve morphological changes in the bicellar structures, that is, from disks to cylindrical micelles to perforated lamellar sheets and mixed multilamellar vesicles [43,63]. Then, a system of small size at physiological temperature that could be use, for instance, for *in vivo* applications would be the DPPC/DHPC *q* = 3.5 bicelles. At 37 °C, below the DPPC *T*_m_, these structures have dimensions of about 15 nm in diameter and 5.4 nm in thickness. These values are consistent with previously reported data of DPPC bilayer thickness [64,65] and bicellar disk dimensions [5,66]

The passage of these small bicelles through some tissues as skin SC seems reasonable, considering that this region is formed by lipid lamellae with narrow interlamellar spaces (between 6 and 10 nm) Coderch 2002 [67]. In fact, the interaction of these systems with SC has been reported and although tracking the presence of the bicellar disks in the tissue was not possible, interestingly, vesicles and lamellar-like structures were observed in the lipid intercellular areas of the tissue after treatment with bicellar systems. These structures were probably the result of a structural rearrangement of bicelles inside the cutaneous SC [55].

DLS and FFEM analyses of the bicellar diluted samples demonstrated the tendency of the bicellar aggregates to grow and form vesicles by the effect of dilution (Figures 12 and 13). It is noteworthy that the phase transitions the bicelles underwent due to the variation of lipid concentration, temperature, and phospholipids molar ratio [68] are very similar to those involved in the reconstitution of the surfactant-lipid micellar systems [69-71]. The transformation of these structures in vesicles was largely discussed in a number of works [72-75]. In earlier studies, we investigated kinetics aspects of this process [76]. A model for the micelle-to-vesicle transition proposed by Leng and co-workers described the rapid formation of disk-like aggregates and their growth and closure to form vesicles [77]. This model took into account line tension dominating bending energy. Certainly, the resemblance of surfactant-lipid micelles and phospholipid bicelles justifies their similar behavior. DHPC solubilizes the DPPC bilayer forming the bicellar structures in a similar way that a surfactant solubilizes lipid vesicles forming micelles. In addition, the high water solubility of DHPC accounts for the structural changes the bicelles underwent. When DHPC is removed from the bicelles upon dilution (*q* increases), the bilayers tend to fuse and the bicellar diameter increases. Morphological changes in the bicelle structure then take place. When sufficient DHPC is removed from the bicelles, the precipitation of large aggregates is visible, that is, phase separation occurs [78].

From these findings, the presence of vesicles in the skin stratum corneum was attributed to a structural transition process from bicelles to vesicles occurring inside of this tissue. The solution used to treat the skin was composed of bicelles with dimensions suitable for stratum corneum penetration. Inside this tissue, bicelles probably transformed in vesicles following a process analogous to that observed in the DLS experiment with diluted bicellar solutions. A similar phenomenon was reported by our group applying octyl glucoside and phosphatidylcholine mixed micelles (OG/PC) in the SC [79]. In this investigation, was demonstrated that liposomes were solubilized by OG and the resulting mixed micelles penetrated through the skin and then were reconstituted in vesicles by effect of the hydration gradient of the tissue.

## 4. Effect of the Inclusion of Ceramides in Bicelles

### 4.1. Type of Ceramides

**Figure 14.**
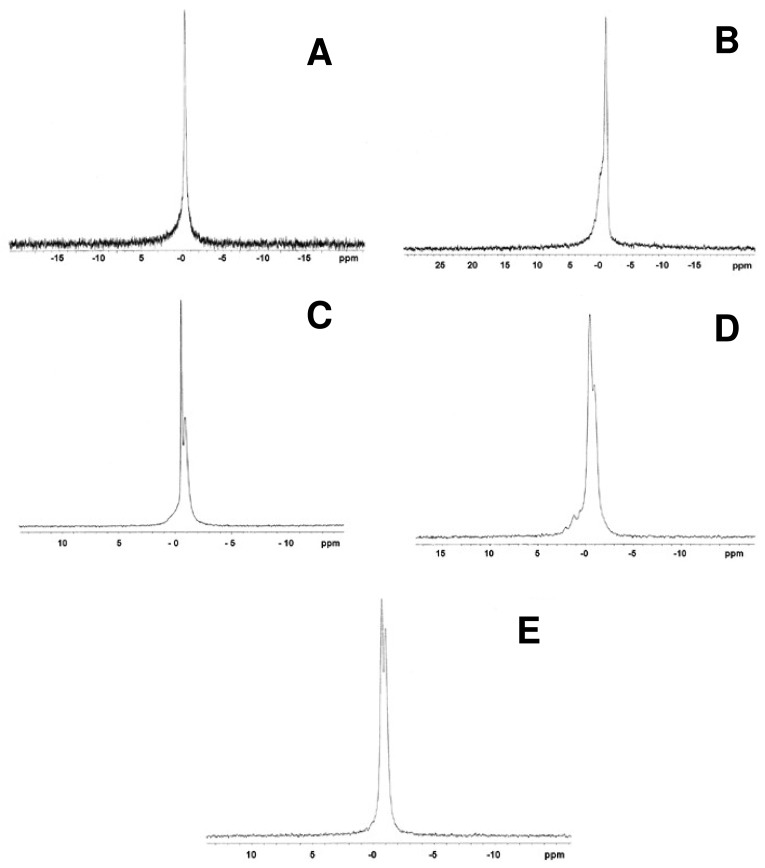
^31^P-NMR spectra at 20 °C of the systems formed by DMPC/DHPC *q* = 2 **(A)**, DMPC/DHPC *q* = 2 + 10% mol C14-Cer **(B)**, DMPC/DHPC *q* = 2 + 10% mol C24-Cer **(C)**, DMPC/DHPC *q* = 2 + 20% mol C14-Cer **(D)** and DMPC/DHPC *q* = 2 + 20% mol C24-Cer **(E)**. Spectra were referenced to 85% phosphoric acid.

The incorporation of two types of Cer in the system DMPC/DHPC *q* = 2, one with alkyl chain of 14 carbons (Cer14) and another with 24 carbon alkyl chain (Cer24) has been reported [19,33]. Both Cer caused structural changes in bicelar systems. The low miscibility between Cer and phospholipids and also the ceramide ability to aggregate to form domains in the bilayer of bicelles was checked [80,81]. In this sense, the technique of ^31^P-NMR has been very useful to confirm the inclusion of Cer in bicellar systems. This inclusion was noted considering changes caused by Cer in the phosphorus spectra from bicelles. Cer14 has proven to be less miscible in bicelles than Cer24, causing a slight influence on the phosphorus signal in the spectra. On the other hand, the inclusion of Cer24 led to a clear differentiation of the phosphorus signal in the spectra, indicating the increased incorporation into systems (Figure 14). Cers have no phosphorus atoms and so do not contribute to the ^31^P NMR signal, but only indirectly through their effects on the phase/mobility/domain state of the phospholipids.

**Figure 15.**
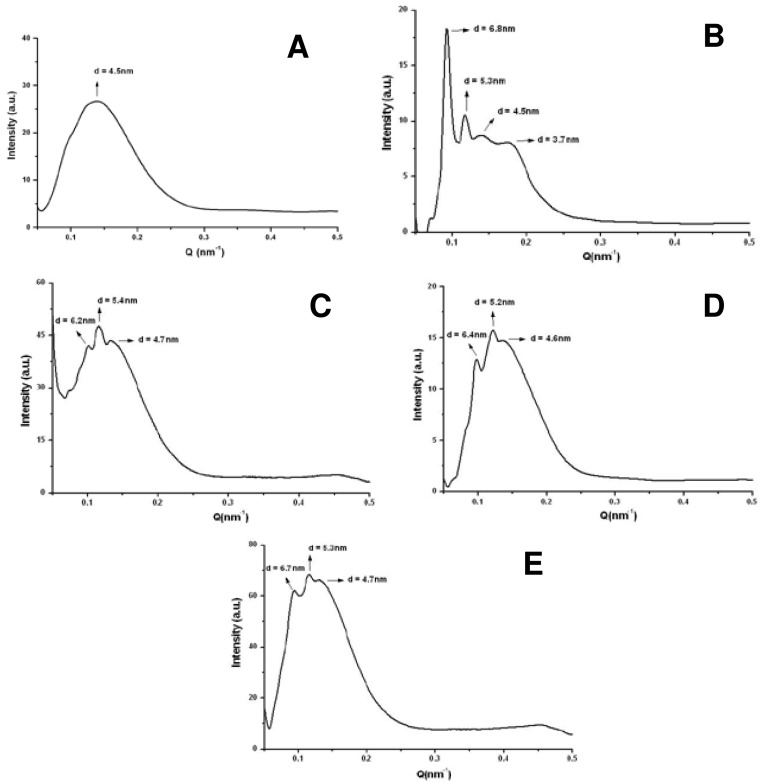
SAXS profiles at 20 °C of the systems formed by DMPC/DHPC *q* = 2 **(A)**, DMPC/DHPC *q* = 2 + 10% mol C14-Cer **(B)**, DMPC/DHPC *q* = 2 + 10% mol C24-Cer **(C)**, DMPC/DHPC *q* = 2 + 20% mol C14-Cer **(D)** and DMPC/DHPC *q* = 2 + 20% mol C24-Cer **(E)**.

The domains formed by Cer in bicellar systems were detected by SAXS (Figure 15). In the spectra several peaks associated with different spacing values (d) were observed. These spaces were attributed to the width of the lipid bilayers of the bicelles [19]. The spacing value for the initial system DMPC/ DHPC (d = 4.5–4.8 nm) was detected in all spectra. In addition to this value, in systems including Cer two values of d, around 5.2–5.4 and 6.2–6.7 nm were obtained. These spacings have been published as related to the formation of different types of ceramide-rich domains that would modify the thickness of the bilayer [80,81].

In the case of the system formed with Cer14, a fourth peak with a value of *d* = 3.7 nm was detected. This value could correspond to non-lamellar structures composed of ceramide, suggesting that this lipid, due to its lower miscibility, probably had not been fully incorporated in the bicelles and it had been segregated. In fact DLS showed a great polydispersity in systems formed with Cer14. Images of this system obtained by FFEM showed rounded aggregates sizing about 15–20 nm and elongated aggregates of approximately 40 nm. This indicated a reorganization of the system with respect to that without Cer, which showed a single type of structure. The Cer14 may have been included in the system irregularly causing the DHPC displacement in some aggregates, thereby increasing the molar ratio (*q*) of these aggregates. This event was reflected in the increased size structures [33]. The displaced DHPC probably settled in the aggregate without Cer, decreasing the molar ratio (*q*) of these aggregates that consequently resulted even smaller than those present in the original system. In fact, the average particle size obtained by DLS for DMPC/DHPC/Cer14 system was about 15 nm, that is, a lower value than that reported for the initial system (about 16 nm) and also lower than the size obtained for DMPC/DHPC/Cer24 system (approx. 19 nm). This system including Cer24 showed homogeneous structures, with particle size slightly larger than the initial system without Cer, indicating that the Cer24 had been incorporated into bicelles [19,33].

### 4.2. Concentration of Ceramides

In order to determine the effect of increasing concentration of Cer on bicelles, systems including 10 and 20% mol of Cer have been studied. At room temperature, we observed that the system could incorporate up to 10% mol of Cer. The inclusion of 20 mol% of this lipid resulted in a destabilization of the structures with the formation of mixed bicelles and vesicles. It has been reported that this destabilization could be observed visually, given that the systems went from transparent to translucent [19]. The membrane destabilization effect of Cer on vesicles has been reported [80,82]. This effect has been ascribed to the high intrinsic curvature of Cer, which gives rise to lateral phase separations [83,84].

**Figure 16.**
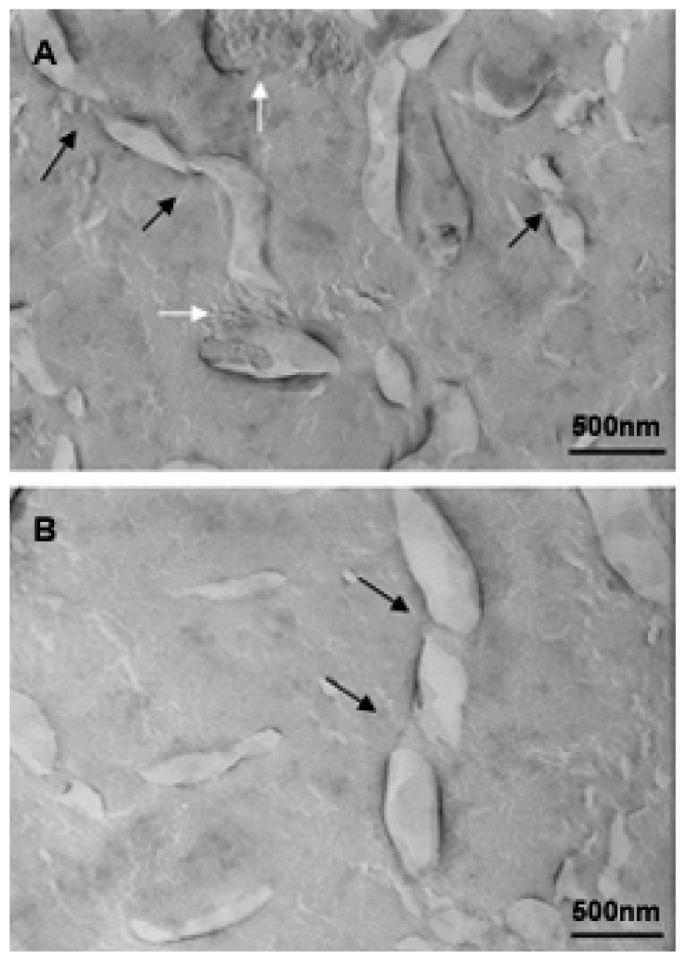
Micrographs of bicellar sample containing Cer at 20 °C. Two different regions of the same sample. Black arrows indicate the twisted zones along the elongated aggregates. The white arrows in image A show the presence of small bicelles in the range of 20–50 nm.

An investigation to determine the influence of temperature on bicellar systems with Cer24 has been carried out. This study, conducted at temperature below (20 °C) and above (40 °C) the *T*_m_ of DMPC (24 °C) and using more sensitive techniques revealed the presence of new structures in the systems [33,83]. In the systems formed with 10% mol of Cer at 20 °C, small bicelles sizing 20 nm and elongated structures twisted at several points were detected (Figure 16).

**Figure 17.**
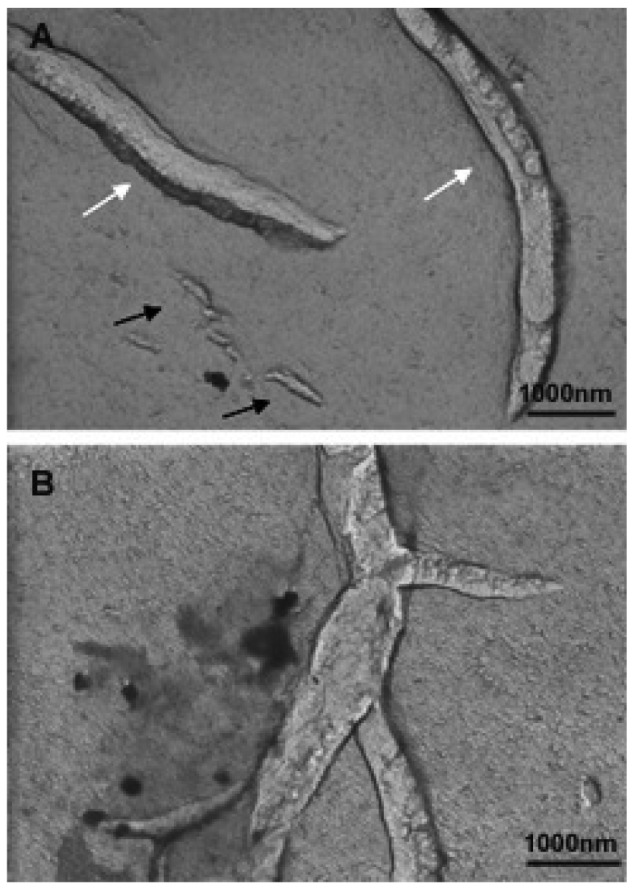
Micrographs of bicellar sample containing Cer at 40 °C. A) and B) represent two different regions of the same sample. In image A the arrows indicate the tubular objects with about 500 nm (black arrows) and 4000 nm (white arrows).

The folded or twisted areas are probably ceramide-rich domains and could be related with the different SAXS peaks showed in Figure 15. Moreover, due to this negative curvature Cer would not pack parallel to their nearest neighbors but rather with a twist angle, which should promote incurvations on the DMPC bilayer. In connection with this, López-Montero *et al.* [85,86] described irregularities in phospholipid membranes as a consequence of asymmetric distribution of Cer between outer and inner monolayers. In those works, asymmetry was induced by the presence of Cer in the external leaflet of giant unilamellar vesicles. In bicellar systems containing Cer, the asymmetry between both leaflets is probable due to the formation of ceramide rich domains. Then, bicelles could twist to relax the surface tension generated by the lipid asymmetry in a similar way to how López-Montero *et al.* described [85]. Increasing the temperature to 40 °C led to the formation of large aggregates, which showed long branches that may have been formed from the folded and/or twisted areas (Figure 17). Such growth would be favored by the movement of DHPC in these areas.

It is interesting to note that the structures with 10% Cer formed at 25 °C, that is, above the *T*_m_ of DMPC did not show large structures as might be expected of systems in fluid liquid crystal state. The structures found in this system were similar to those found in gel-state systems that are observed in different works [32,33,47,87]. This is explained by the fact that Cer induce the gel phase in DMPC systems [81,88], which extends the temperature range in which these systems would be in the gel phase.

It is also curious that systems at 20 °C presented greater structural diversity than systems at 25 °C [19,33]. This fact could be explained based on the sensitivity of the techniques used in these two experiments. The particle sizes of the systems studied at 20 °C and 40 °C were measured with a Nanosizer Nano ZS. This is an appropriate tool to study bicellar systems due to its ability to measure concentrated samples without dilution. The 4700c Malvern apparatus used to measure the samples at 25 °C in a previous work [19] is more suitable for diluted samples. In addition, the HPF used for samples at 20 °C and 40 °C [33] is also advantageous to the fixation by propane immersion used in the experiment at 25 °C [19]. Although none of the cryofixation methods used in the mentioned articles involves chemical treatment or dilution of samples, HPF is especially appropriate because the samples are frozen under optimal conditions of temperature and pressure, and changes in the structure of systems are avoiding. Thus, the implementation of high pressure fixation and use of Nanosizer with samples at 20 °C and 40 °C provided higher quality measures and, therefore, higher structural diversity could be detected in the samples.

In the systems formed with 20% mol of Cer, structures of various sizes and shapes were observed at all temperatures studied. The temperature increase, inducing the transition from gel phase to the fluid liquid crystal phase, promoted formation of multilamellar liposome and tubular structures coexisting with small bicelles. At this concentration, Cer are not incorporated completely in the systems and this fact cause destabilization of the systems.

## 5. New Strategies Based on Bicellar Systems

Bicelles constitute very promising systems for different applications. This review shows that such systems exhibit intelligent properties, since they respond to external stimuli. Its versatility, the ability to modulate its structure, organization and their lipid bilayer offer great advantages over other bilayer systems. Besides, some works have demonstrated that these systems are adequate to penetrate the skin and to reinforce the structure lipid system (DPPC / DHPC *q* = 3.5) or to enhance the permeability of this tissue (DMPC / DHPC *q* = 2) [47,55,87].

Structural characterization of bicelles has revealed interesting aspects about the behavior of these systems and about the different effect induced by inclusion of skin lipids, such as ceramide. In this way, the utility of bicelles as membrane model to study skin lipids could be considered in future research.

With regard to the use of bicellar systems as vehicles for skin delivery, the entrapping ability of bicelles is being evaluated [89]. Systems containing Cer and dichlofenac diethylamine have been analyzed by DLS and Cryo-TEM [89]. Systems including lipophilic compounds were found to be larger than the initial systems, suggesting that these actives may be incorporated in the bilayers of the bicellar structures. On the other hand, the inclusion of hydrophilic actives caused a reduction in size and a rounding of bicellar structures. This fact could be explained by the surfactant nature of the hydrophilic compounds includes, which have more affinity for incorporation into the edges of the bicelles, decreasing the molar ratio of the systems, a fact that would cause the decrease in the size of the aggregates.

Although the characteristic of bicelles to change structurally and morphologically depending on the environmental conditions allows for its use in different areas, this property may limit its application in environments where these conditions are variable. Bicelles can be considered good carriers for the skin. However, their application as carriers for administration through the systemic route, where the water content is high, would represent a challenge, as well as its simple addition to vehicles with higher water content. At diluted conditions bicelles properties would be lost and the systems would present variable morphologies and effects.

**Figure 18.**
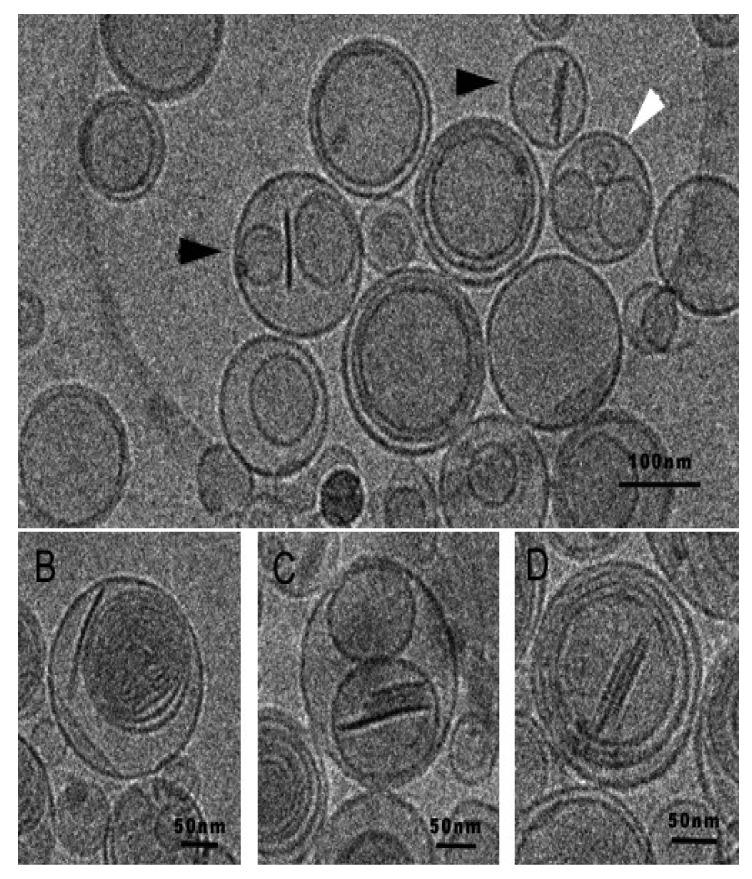
Cryo-TEM images of a sample containing bicosomes. **(A)** High variability of vesicles is observed: empty vesicles (black arrows); oligolamellar structures (white arrowheads) and multilamellar vesicles (white arrows); vesicles with bicelles inside (black arrowheads). **(B)** Multilamellar vesicles. **(C)** Stacked bicelles inside vesicles. **(D)** Stacked bicelles inside multilamellar vesicles.

In order to address this limitation, Rodriguez *et al.* have recently proposed a strategy to preserve the discoidal morphology of bicelles for its use in high water content environments. This strategy consists in the preparation of new structures also formed by phospholipids, the so-called bicosomes that would protect bicelles from dilution, preserving the aggregates until the target tissue is achieved [90]. Cryo-TEM images of bicosomes are shown in Figure 18.

Other methods have been used to stabilize the morphology of discoidal bilayers, such as using bicelles with charged amphiphiles [91] or disks formed by mixtures containing polyethylene glycol-lipid conjugates [92]. However, with the use of PEG-lipids, the properties of bicelles related to structural versatility, such as the enhancer effect of the permeability on some physiological barriers, could be lost.

## 6. Conclusion

All in all, bicellar systems are emerging as a fascinating, morphologically versatile lipid nanostructure that open up new possibilities of study and applications owing to their size, shape and modulable structure. These bicellar systems could be useful as membrane mimics for studies in which Cer are involved. Differences in cer structure and concentration give rise to diverse effects on bicelles. The systems are able to incorporate 10% mol of Cer, which is probably organized in domains along the bilayer. The addition of 20% mol promotes destabilization of the discoidal bicelles, resulting in the formation of mixed systems with bigger structures. Results presented prove that it is possible to create a model membrane made up of bicelles including Cer. Additionally, the absence of surfactant in their exclusively lipid composition, and the possibility of including different actives, makes these systems a good candidate as colloidal carriers. Moreover, the systems *per se* are able to induce specific effects on the skin and the possibility of this being addressed for other tissues appears to pose another interesting option for the near future.
